# Gene Expression Profiling of Cutaneous Injured and Non-Injured Nociceptors in SNI Animal Model of Neuropathic Pain

**DOI:** 10.1038/s41598-017-08865-3

**Published:** 2017-08-24

**Authors:** Temugin Berta, Florence E. Perrin, Marie Pertin, Raquel Tonello, Yen-Chin Liu, Alexander Chamessian, Ann C. Kato, Ru-Rong Ji, Isabelle Decosterd

**Affiliations:** 1 Pain Center, Department of anesthesiology, Lausanne University Hospital (CHUV) and Faculty of biology and medicine (FBM), University of Lausanne (UNIL), 1011 Lausanne, Switzerland; 2 Department of Anesthesiology, Duke University Medical Center, 595 LaSalle Street, Durham, NC 27710 USA; 3 Pain Research Center, Department of Anesthesiology, University of Cincinnati Medical Center, Cincinnati, Ohio, 45267 USA; 4 Department of Basic Neuroscience, Faculty of Medicine, 1211 Geneva 4, Geneva, Switzerland; 5 University of Montpellier, Montpellier, F-34095 France, INSERM, U1198, Montpellier, F-34095 France, EPHE, Paris, F-75014 France; 6 Department of Anesthesiology, College of Medicine, National Cheng Kung University, Tainan city, Taiwan; 7 Department of Fundamental Neurosciences, Faculty of biology and medicine (FBM), University of Lausanne (UNIL), 1005 Lausanne, Switzerland

## Abstract

Nociceptors are a particular subtype of dorsal root ganglion (DRG) neurons that detect noxious stimuli and elicit pain. Although recent efforts have been made to reveal the molecular profile of nociceptors in normal conditions, little is known about how this profile changes in pathological conditions. In this study we exploited laser capture microdissection to specifically collect individual injured and non-injured nociceptive DRG neurons and to define their gene profiling in rat spared nerve injury (SNI) model of neuropathic pain. We found minimal transcriptional changes in non-injured neurons at 7 days after SNI. In contrast, several novel transcripts were altered in injured nociceptors, and the global signature of these LCM-captured neurons differed markedly from that the gene expression patterns found previously using whole DRG tissue following SNI. Pathway analysis of the transcriptomic profile of the injured nociceptors revealed oxidative stress as a key biological process. We validated the increase of caspase-6 (CASP6) in small-sized DRG neurons and its functional role in SNI- and paclitaxel-induced neuropathic pain. Our results demonstrate that the identification of gene regulation in a specific population of DRG neurons (e.g., nociceptors) is an effective strategy to reveal new mechanisms and therapeutic targets for neuropathic pain from different origins.

## Introduction

Dorsal root ganglion (DRG) sensory neurons are specialized for the detection of various somatosensory stimuli including touch, temperature and pain. Different stimuli are decoded by different subsets of sensory neurons with distinct receptors, ion channels and signaling molecules. Despite this functional and molecular heterogeneity, transcriptional profiling of sensory neurons has focused on analyzing the whole DRG tissue^1–6^.

In the past years, there has been significant progress in revealing molecular signatures of different DRG neuronal subtypes subserving different sensory modalities. For instance, the use of single-cell analysis of dissociated DRG neurons captured by glass capillary has characterized eleven distinct subtypes of sensory neurons for somatosensation^7, 8^. Similarly, the optimization of cell sorting and use of transgenic mice has revealed distinct expression patterns of neurons specifically involved in nociception and proprioception^9^. Although these studies have significantly expanded our knowledge of different neuronal DRG subtypes and molecular signatures underlying somatosensation, they cannot retain gene expression profiles in intact ganglia with anatomical features of different neurons, and dissociated DRG neurons in cultures are known to change their gene expression profiles in part due to axonal injury during dissociation (e.g., ATF3^10, 11^). Furthermore, all these studies did not address the molecular changes in DRG neurons in pathological conditions such as after nerve injury.

Nociceptors are a particular subtype of DRG sensory neurons specialized to detect painful stimuli^12^. Like most of the peripheral sensory neurons, nociceptors have their cell bodies in the DRG, give rise to an unmyelinated or thinly myelinated axon that bifurcates into a peripheral branch that innervates peripheral target tissues, and have a central branch enters the dorsal horn of the spinal cord. Nociceptors react to external stimuli by adaptive responses that enable our body to avoid potential and actual damages. However, when the peripheral axon of nociceptors is severed, profound transcriptional changes occur and produce maladaptive function that can drive neuropathic pain.

Neuropathic pain affects up to 10% of the worldwide population and is associated with risk factors that include mental state, gender, age, and anatomical site of injury^13^. Peripheral nerve injury can lead to neuropathic pain, so that pain occurs spontaneously, and responses to painful and innocuous external stimuli are pathologically amplified. To better understand the pathological processes underlying this condition, we combined the selective isolation of injured and non-injured nociceptors by laser capture microdissection (LCM) with microarray analysis in the spared nerve injury (SNI), an animal model of neuropathic pain. Nociceptor-specific gene profiling showed minimal transcriptional changes in non-injured neurons suggesting translational changes as explanation of their ectopic firing and contribution to neuropathic pain^14^. Interestingly, the transcriptional analysis of injured nociceptors revealed a significantly different signature from whole DRG tissue gene profiling and the early regulation of genes involved oxidative stress. In addition, we identified a prominent role of caspase-6 and evaluated its potential as a therapeutic target to alleviate neuropathic pain.

## Results

### Laser-capture microdissection of cutaneous nociceptors in SNI animal model of neuropathic pain

**Figure 1:**
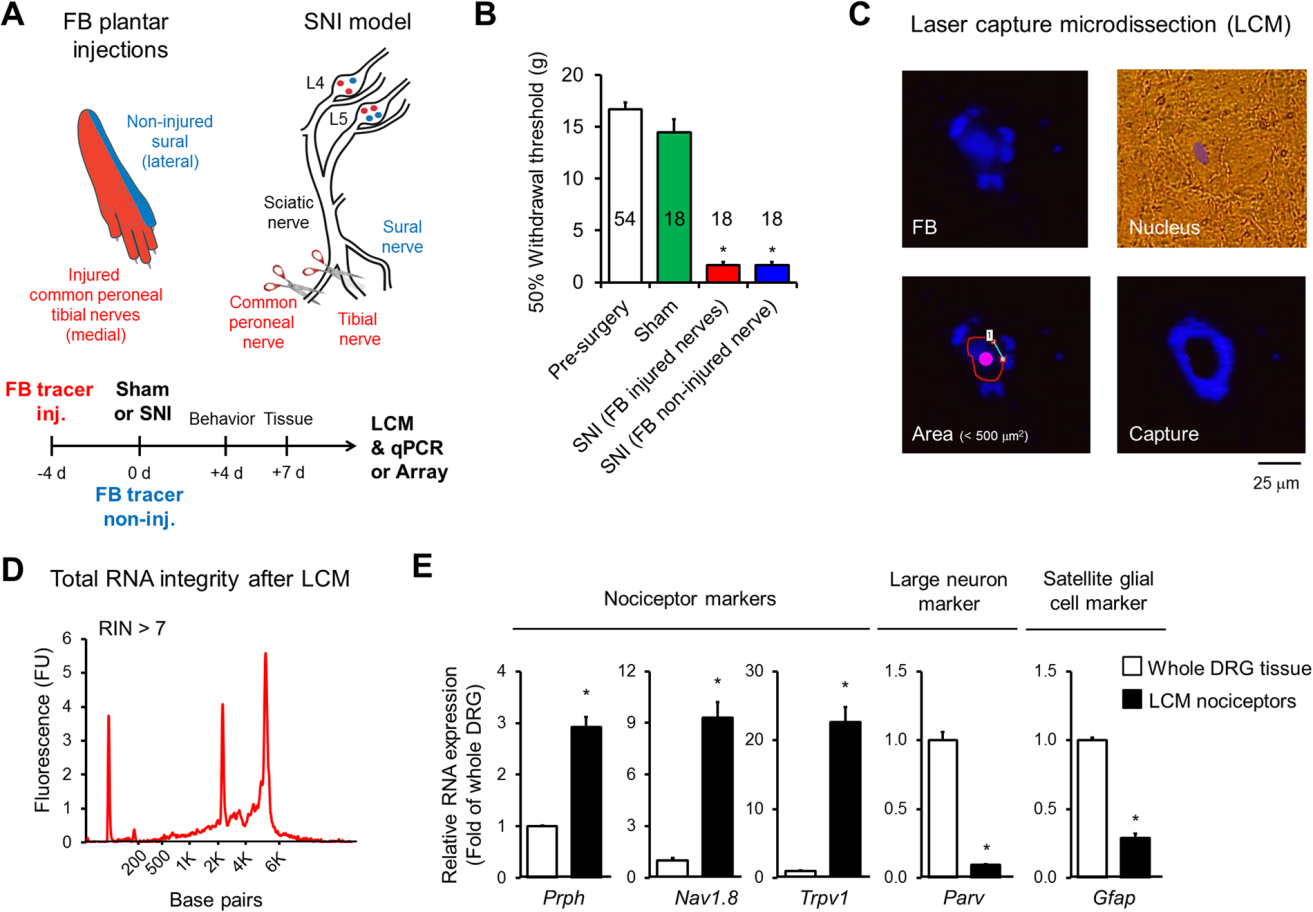
Laser capture microdissection (LCM) of injured and non-injured nociceptors in spared nerve injury (SNI) model of neuropathic pain. (**A**) Schematic of experimental procedures. Fast blue (FB 2%) was injected in tibial/common peroneal nerves or sural nerve skin territories for retrograde tracing of injured and non-injured DRG neurons, respectively. Sham animals received FB in all territories. L4 and L5 DRG tissues were collected 7 days after SNI or sham surgery. Tissues from 54 animals were used for LCM (18 per group: sham, SNI - FB injured nerves, and SNI - FB non-injured nerve. (**B**) All SNI rats developed a robust mechanical hypersensitivity tested on day 4 after surgery. (**C**) Representative microphotographs of microdissected DRG neurons. Only small-sized neurons (cross-sectional area <500 μm^2^) with presence of FB and nucleus were microdissected from DRG tissue sections. (**D**) The Agilent LabChip technology was used to control the quality of the RNA and only samples with high-quality total RNA (RIN >7) were used for qRT-PCR or microarray analyses. (**E**) To confirm the specific capture of nociceptors, we compared by qRT-PCR the expression of neuronal and glial markers in LCM captures nociceptors (black bars) to whole DRG tissue (white bars) (*p < 0.05, t-test, n = 3). To note typical nociceptive markers, such as *Na*_*v*_ *1*.*8* and *Trpv1*, were all significantly increased in LCM samples.

To study the transcriptional responses of specific nociceptors in chronic pain, we used the spared nerve injury (SNI) animal model of neuropathic pain, wherein the tibial and common peroneal nerves are ligated and transected while the sural nerve is left intact (Fig. 1A). Because molecular and functional changes in both injured and non-injured nociceptors contribute to the development of neuropathic pain, we injected the retrograde tracer Fast Blue (FB) in the hind paw region of the tibial and common peroneal nerves 4 days before the surgery or in the region of spared sural nerve immediately after the surgery to distinguish the cell bodies of the respective nerves in the dorsal root ganglia (Fig. 1A). The injection in the region of spared sural nerve was performed after the SNI surgery (axotomy of the injured nerves) in order to impair any unintentional retrograde labelling of the injured neurons. The development of neuropathic pain was assessed by the presence of mechanical hyperalgesia in all animals used for transcriptional analysis 4 days after surgery (Fig. 1A and B). All SNI animals had significantly reduced thresholds to mechanical stimuli in the ipsilateral hind paw, whereas sham animals showed no difference comparded to their pre-surgery baseline (Fig. 1B). Injured and non-injured neuronal cell bodies were individually isolated by laser-capture microdissection (LCM) from dorsal root ganglia sections according to the following criteria: (1) the presence of FB fluorescence, (2) an identifiable nucleus and (3) a cross-sectional area <500 μm^2^ (Fig. 1C and Suppl. Mov. 1). A cross-sectional area of area <500 μm^2^ ensured the collection of less than 5% of large-sized and neurofilament NF200 positive DRG neurons and more than 90% of peripherin positive neurons, a protein that is expressed by the majority of small-to-medium-sized DRG neurons^15^ (Suppl. Fig. 1). Applying these criteria, we collected by LCM about 400 neurons per animal from L4/L5 DRG tissues and collected neurons from 6 animals were pooled (an average of 2463 ± 124 neurons) to obtain sufficient material for the following quality tests and microarray analysis. Prior to microarray analysis, total RNA was extracted and its quality was assessed by electrophoresis. Only high quality RNA showing clear 18 S and 28 S ribosomal peaks and with a 28 S/18 S area ratio higher of 1.7 and a RIN >7 were retained and proceeded for the microarray analysis (Fig. 1D). We have also verified the specificity of the laser-capture microdissection by comparing the captured nociceptor and whole DRG transcriptional expression of well-known neuronal and glial markers (Fig. 1E). Nociceptor markers (*Prph*, *Nav1*.*8*, and *Trpv1*) were highly enriched following LCM, whereas the large-sized neuron (*Parv*) and glial markers (*Gfap*) were low. The specificity and the enrichment of nociceptors in our samples of the laser capture microdissection technique was also confirmed by the high expression of several nociceptor-specific transcripts in the microarray data from the LCM neurons of sham animals (e.g. *Cgrp*, *P2×3*, *Trpv1*, *Nav1*.*8*, *and Nav1*.*9*) (Suppl. Fig. 2 and Suppl. Table 1).

### Gene expression profiling in injured and non-injured cutaneous nociceptors after SNI

**Figure 2:**
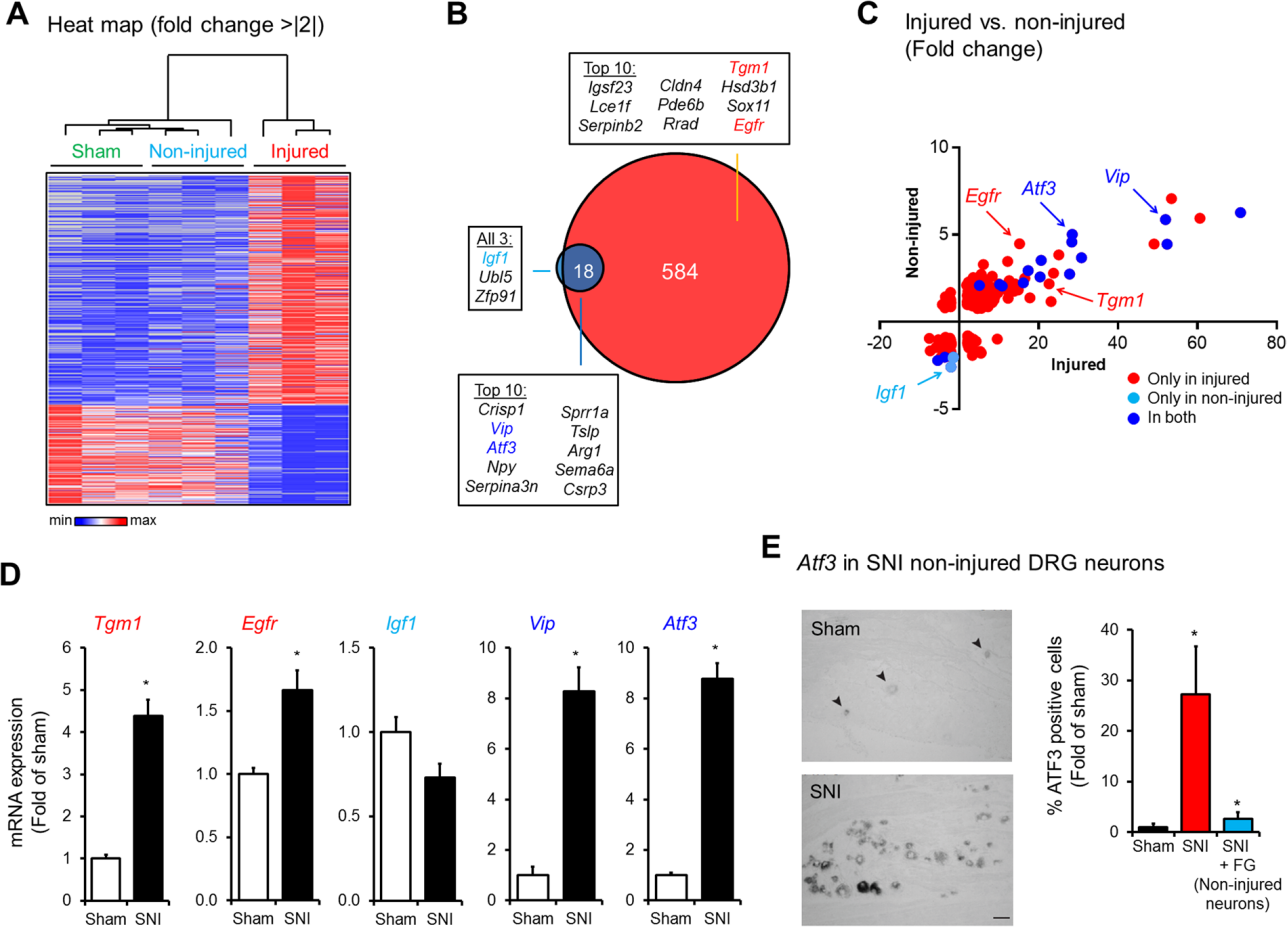
Gene expression profiling of injured and non-injured nociceptors after SNI. (**A**) Heat map of the significantly deregulated genes in injured and non-injured nociceptors compared to sham (Mann –Whitney pair-wise comparison test, fold change > |2|, n = 3) (**B**) Differentially regulated genes in injured (red) and non-injured (blue) nociceptors after SNI displayed as Venn diagram with the top deregulated genes in each group (from Suppl. Table 3). (**C**) XY graph representing the fold changes of all significantly deregulated genes in either injured (red) or non-injured (light blue) or both (dark blue) nociceptors after SNI. (**D**) Validation of microarray data for *Tmg1*, *Egfr*, *Igf1*, *Vip* and *Atf3* by qRT-PCR in SNI versus Sham (*p < 0.05, t-test, n = 4–6). (**E**) Increase in *Atf3* mRNA expression in non-injured neurons was confirmed by combining the retrograde tracing of non-injured neurons with fluorogold (FG) and *in situ* hybridization (*p < 0.05, t-test, n = 4, scale bar = 50 μm).

A total of 9 LCM samples, collected and pooled from 54 animals (6 animals per sample and 3 samples per condition – sham neurons, SNI non-injured and SNI injured neurons), were analyzed by microarray. Heat map and hierarchal clustering of differentially expressed transcripts through the different samples clearly segregated injured nociceptor samples into a separate cluster from sham and non-injured nociceptor samples (fold change >|2| and pairwise comparison >77%, see materials and methods) (Fig. 2A). Out of 31,099 probe sets examined on the arrays, 21 transcripts (<0.01% of total) in the non-injured nociceptors and 602 transcripts (~2% of total) in the injured nociceptors were determined to be differentially expressed in SNI animals compared to sham control and ~85% of the transcripts altered in the non-injured nociceptors are in common with the injured nociceptors (Fig. 2B and Suppl. Table 2). Most of these transcripts were up-regulated (Fig. 2C and Suppl. Fig. 3A) and *Tgm1*, *Egfr*, as well as *Vip* were confirmed by qRT-PCR of L4/L5 DRG tissues (Fig. 2D). We note that the regulation of *Igf1*, which is significantly down-regulated only in the non-injured neurons, was not confirmed by qRT-PCR of L4/L5 DRG tissues (Fig. 2D). In contrast, we observed an increase of *Atf3* in the microarray data sets from the non-injured neurons (Fig. 2B and C), which was confirmed by qRT-PCR of L4/L5 DRG tissues (Fig. 2D). This observation is at odds with the report and general use of ATF3 as a conventional marker for injured neurons^11^. To confirm the increase of *Atf3* in non-injured neurons, we analyzed its expression in L4/L5 DRG tissue after SNI by *in situ* hybridization in combination with the injection of Fluorogold (FG) retrograde tracer in the hindpaw regions innervated by the intact sural nerve^16^. Similar to our microarray finding, *Atf3* is increased in L4/L5 DRGs from 7 d SNI animals (59.1 ± 17.4%, p < 0.05) over sham animals (2.2 ± 1.5%), but *Atf3* is also significantly increased, although to a much lesser extent, in FB positive the non-injured neurons after SNI (5.6 ± 3.0%, p < 0.05) (Fig. 2E). Thus, taken together, our data corroborate studies indicating that *Atf3* is slightly expressed and potentially involved in pain-related mechanisms in non-injured neurons^17–19^. To note, ATF3 has also been associated with neuronal stress^20^ and the transcriptional increase of *Atf3* in non-injured neurons in response to stressful conditions.

### Microarray displays different gene expression profiles from DRG nociceptors and whole DRG tissue after SNI

**Figure 3:**
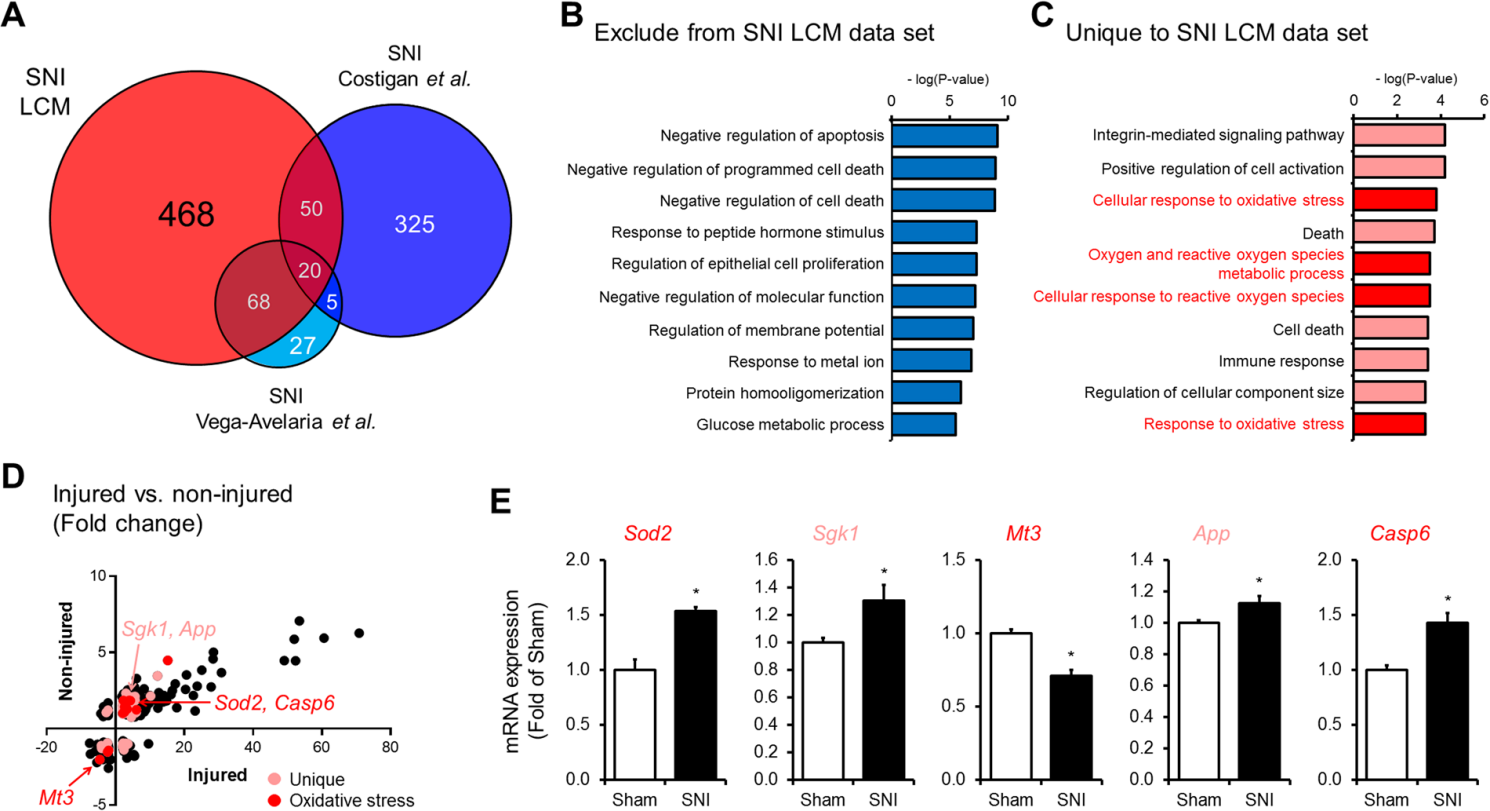
Identification of specific pathways and genes in nociceptors after SNI by transcriptome comparisons with similar previous studies using DRG whole-tissues. (**A**) Venn diagram representing the comparison of the deregulated genes generated by LCM of nociceptors with whole-tissue studies of Costigan *et al*.^21^ and Vega-Avelaria *et al*.^1^ after SNI. To note almost 80% of the deregulated genes detected in the LCM data set have not been detected by these previous studies. (**B**) Functional classification of over-represented genes deregulated obtained by the exclusion of LCM data set or (**C**) unique to this data set (top 10 Gene Ontology categories). (**D**) XY graph showing the fold changes of all the deregulated genes in injured and non-injured nociceptors after SNI that are unique to the LCM data set (pink) and related to oxidative stress (red). (**E**) Validation of unique and oxidative stress related-genes *Sod2*, *Sgk1*, *Mt3*, *App* and *Casp6* by qRT-PCR (*p < 0.05, t-test, n = 4–6).

We next compared the transcriptional changes induced in rats by SNI at 7 day in LCM collected nociceptors with those observed in DRG whole tissue^1, 21^. We found that only 138 genes of the 606 regulated genes in the LCM data set overlap with differentially regulated genes in whole DRG tissue analyses (Fig. 3A). Common neuronal transcripts include those well known for their roles in nociception such as neurotransmitters *Vip*, *Tac1*, *Npy*, as well as neuronal ion channels such as *Scn10a* and *Scn11a* (Suppl. Table 3). Interestingly, neuronal transcripts regulated in whole tissue analyses but not present in the LCM data set include Nefh, Trk3, Scn8a and Pavlb (Suppl. Table 3). These transcripts are characteristically expressed in large sensory neurons^9^ highlighting the specificity of our LCM collection of nociceptors. In general, this data set is associated with survival, proliferation and glucose functions (Fig. 3B). In contrast, genes that are unique to the LCM data set are mostly characterized with functions associated with oxidative stress (Fig. 3C). Genes unique to the LCM data set and associated to oxidative stress were confirmed by qRT-PCR (Fig. 3D). Out of 6 genes tested we were able to validate the expression levels of *Sod2*, *Sgk1*, *Mt3*, *App*, *Casp6* (Fig. 3E), but not *Duox2* (inconsistent standard curve, data not shown). Of particular interest is the regulation of *Casp6* because its implications in axonal degeneration of sensory neurons^22^ and in pain hypersensitivity^23^, two hallmarks of neuropathic pain.

### Caspase 6 is expressed in human and rodent DRG neurons and its inhibition decreases SNI-induced mechanical allodynia

**Figure 4:**
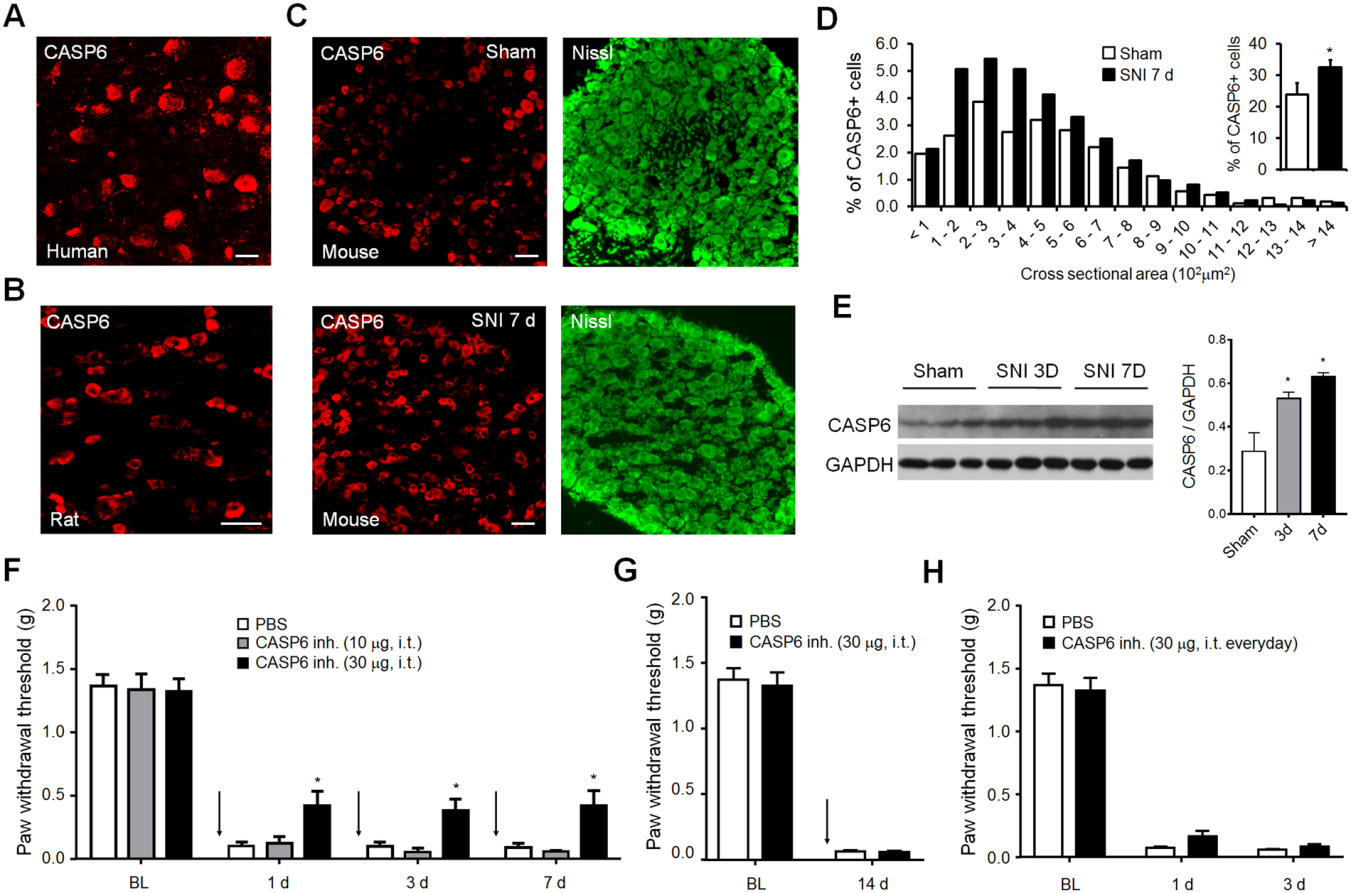
Caspase-6 (CASP6) expression in DRG tissue and deregulation after SNI. Immunohistochemistry indicates the presence of CASP6 in DRG neurons of (**A**) human and (**B**) rat (scale bar = 50 μm). (**C**,**D**) CASP6-like immunoreactivity is increased in small-to-medium DRG neurons ( < 700 μm^2^) 7d after SNI compared to sham (*p < 0.05, t-test, n = 4). Nissl stain was used to characterize the neuronal morphology. Scale bars in panels A, B and C is 50 μm. (**E**) Western blot analysis showing the bands of CASP6 (35 kDa) 3 d and 7 d after SNI and quantification of the intensity of CASP6 bands normalized by the expression of GAPDH (full-length blots/gels, n = 3, one-way ANOVA: F (2, 6) = 10.93, p = 0.01, Bonferroni’s multiple comparisons test *p < 0.05). (**F**) SNI-induced mechanical allodynia is significantly reduced in mice receiving intrathecal (i.t.) injection of 30 μg of CASP6 inhibitor (CASP6 inh.) at day 1, 3 and 7 after SNI (n = 6 mice/group, two-way ANOVA for treatment: F (1, 10) = 6.64, p = 0.0276, Bonferroni’s multiple comparisons test *p < 0.05). No significant effect was observed in mice receiving a lower dose or PBS as vehicle control. (**G**) SNI-induced mechanical allodynia is unchanged in mice receiving i.t. injection of 30 μg of CASP6 inh. at 14 d after SNI. (**H**) SNI-induced mechanical allodynia is not prevented by daily i.t. injection of 30 μg of CASP6 inh. from day -1 to day 3 after the SNI surgery. Mechanical allodynia was tested just before the daily injection of CASP6 inh. (for the behavioral experiments in panel G and H, two-way ANOVA, n = 6 per group).

To validate our transcriptional data, we also performed immunohistochemistry to examine whether the CASP6 protein is present in primary sensory neurons. In sections of human, rat as well as mouse lumbar DRGs, immunoreactivity for anti-CASP6 was strongly expressed in sensory neurons (Fig. 4A–C). Immunohistochemistry with Nissl stain to label the neuronal cytoplasm of all DRG neurons showed the predominant presence of CASP6 in small-to-medium murine sensory neurons (Fig. 4C and D) and CASP6 was expressed by 23.9 ± 3.7% DRG neurons in sham animals and by 32.3 ± 2.5% DRG neurons after 7 day SNI (Fig. 4D). This increase in CASP6 protein expression was also validated by Western Blot (Fig. 4E). To test the functional role of CASP6 signaling in SNI, mice were injected intrathecally with vehicle control or the specific CASP6 inhibitor Z-VEID-FMK (10 and 30 µg) at day 1, 3 and 7 after SNI (Fig. 4F). The high dose of Z-VEID-FMK significantly reduced the mechanical allodynia in mice tested 1 h after the intrathecal injection (Fig. 4F). However, the Z-VEID-FMK effect disappears at day 14 after SNI (Fig. 4G), suggesting a role of CASP6 in the development but not in the maintenance of the mechanical allodynia. We have also tested whether CASP6 inhibition was involved in the prevention of the SNI-induced mechanical allodynia. Daily injections of CASP6 inhibitor form 1 day before up to 3 days after the SNI surgery were found ineffective to prevent the appearance of the mechanical allodynia after SNI (Fig. 4H). These results indicate that CASP6 signaling is involved in the reversal but not in the prevention of the development of neuropathic pain.

### Attenuation of paclitaxel-induced neuropathic pain in Casp6 knockout mice

**Figure 5:**
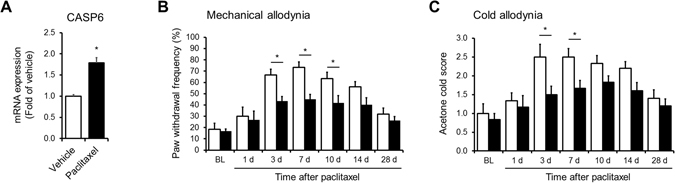
CASP6 contributes to the development of neuropathic pain induced by the chemotherapeutic drug paclitaxel. (**A**) qRT-PCR analysis showing mRNA expression levels of CASP6 in vehicle vs. paclitaxel injected animals (*p < 0.05, t-test, n = 4). (**B**) Mechanical hypersensitivity expresses as frequency response to 0.4 g von Frey hair stimulation in wild-type (WT. white bars) and caspase-6 knockout (Casp6^−/−^, black bars) animals (n = 5–6 mice/group, two-way ANOVA for genotype: F (1, 66) = 22.01, p < 0.0001, Bonferroni’s multiple comparisons test *p < 0.05). (**C**) Cold allodynia in wild-type (WT, white bars) and caspase-6 knockout (Casp6^−/−^, black bars) animals (n = 5–6 mice/group, two-way ANOVA for genotype: F (1, 66) = 16.95, p < 0.0001, Bonferroni’s multiple comparisons test *p < 0.05).

Next, we sought to test the functional significance of the CASP6 in a pathological pain model associated with oxidative stress. The chemotherapeutic paclitaxel induces mitochondrial dysfunction and oxidative stress in nociceptors that results in mechanical and cold allodynia^24, 25^. Similarly to SNI, paclitaxel induced a significant increase of the CASP6 transcript 7 days after treatment (Fig. 5A). More importantly, both paclitaxel-induced mechanical and cold allodynia were significantly reduced in Casp6 knockout (Casp6^−/−^) mice compared to wild-type (WT) mice (Fig. 5B and C), indicating a contribution of CASP6 toward the paclitaxel-associated neuropathic pain.

## Discussion

It is well-reported that injured and non-injured DRG neurons contributes to neuropathic pain^26^. High-throughput transcriptional analyses have been mostly limited to individual dissociated DRG neurons from naïve animals or at the whole DRG tissue in various neuropathic pain conditions. Although these analyses have been incremental to the classification of various neuronal subpopulations and our general understanding of neuropathic pain, very little is known about the comprehensive transcriptional changes occurring in discrete neuronal subpopulations in neuropathic pain conditions. In particular nociceptors undergo maladaptive functional and phenotypic changes in response to peripheral injury that can results in neuropathic pain^12, 27, 28^.

In the present study, we uncovered the specific transcriptional signatures of injured and non-injured nociceptors in the SNI model of neuropathic pain using laser-capture microdissection coupled with microarray analysis. Using this approach, we were able to isolate injured and non-injured cutaneous nociceptors from DRG tissues with minimal processing and contamination from other surrounding neuronal and glial cells (Fig. 1E). As a result, we identified the regulation of several novel genes and pathways in cutaneous nociceptors that were undetected or masked by previous conventional analyses of whole DRG tissues in neuropathic pain (Fig. 3A, Suppl. Table 3). Using pathway analysis, we found oxidative stress to be robustly activated by nerve injury. Consistent with the predictions of this analysis, we implicated CASP6, a key player in the oxidative stress pathway, in the pathogenesis of neuropathic pain. In addition, we revealed the involvement of CASP6 into another model of neuropathic pain where toxicity of paclitaxel is involved in nerve injury. Thus, we now propose CASP6 as new therapeutic target (Figs 3C and 5).

Unlike previous microarray analyses that studied whole DRG tissues after SNI, we collected and exclusively analyzed cutaneous injured and non-injured nociceptors by the injection of the retrograde tracer Fast Blue in the hindpaw (Fig. 1A). Cutaneous nociceptors are currently classified into three major functionally-distinct types: peptidergic nociceptors, non-peptidergic nociceptors and C-low-threshold mechanoreceptors (C-LTMRs)^28, 29^. Our microarray data from sham animals display the expression of gene markers of peptidergic (e.g. *Calca* and *TrkA*) and non-peptidergic nociceptors (e.g. *P2rx3* and *Ret*), but not of C-LTMRs (e.g. *Th* and *Vglut3*), suggesting that the majority of our collected neurons were indeed nociceptors (Suppl. Fig. 2). This is not surprising since we injected retrograde tracer subcutaneously in the glabrous skin of the hindpaw and one of the most striking features of C-LTMRs is that they are only found in hairy skin^30^. Although we detected several mRNAs characteristic of nociceptors in our microarray analysis, other specific mRNAs were missing such as *Trpa1*, *Trpm8* and *MrgprD* ^9^. This is probably due to the technical limitations of microarray (e.g., probe and hybridization efficiency) because, for instance, we were able to observe an increased Trpa1 by qRT-PCR in our LCM captured cells compared to whole DRG samples (data not shown).

In the SNI model, pain hypersensitivity is observed in the territory supplied by the spared sural nerve of the hindpaw skin^31^. Functional plasticity of the intact nociceptors in the sural territory has been demonstrated, providing a mechanistic foundation for the pain behaviors observed in this model^32^. Such robust changes at the behavioral and electrophysiological levels suggest a common origin in altered gene expression. However, our microarray data do not indicate major transcriptional changes in non-injured neurons after SNI (Fig. 2A and B).This surprising finding implies that post-transcriptional processes may be the predominant drivers of functional plasticity of intact neurons in the neuropathic state. Indeed, we and others have shown previously that nerve injury induces robust changes in the nearby intact nerves at the protein level but without a corresponding change at the mRNA level. For example, we demonstrated that the voltage-gated sodium channel β2 subunit, which is known to contribute to mechanical allodynia, is significantly upregulated in non-injured neurons after SNI at the protein level only^14^. Through its role in regulating the transport of voltage-gated sodium channels (e.g. Na_v_1.7) and modulating channel gating, the β2 subunit may play a key role in the post-transcriptional processes in non-injured sensory afferents and, thereby, neuropathic pain^14^. A change in protein turnover rate of voltage-gated sodium channels by decreasing protein degradation by the lysosomal proteolytic pathway could also be prominent post-transcriptional regulations in neuropathic pain^33^. However, whether this mechanism occurs in both injured and non-injured neurons is unclear.

Although our findings are consistent with this body of evidence, due to the inherently limited sensitivity of microarrays, we cannot exclude the occurrence of important transcriptional changes in the sural nerve. Indeed, other, more sensitive methods of gene expression analysis, such as real-time qRT-PCR and *in situ* hybridization, have revealed the up-regulation of algesic neuropeptides, growth factors and ion channels (e.g. CGRP, BDNF and TRPA1) in non-injured neurons after partial nerve ligation (PSL) and spinal nerve ligation (SNL)^34–37^. Similar transcriptional changes may occur in the intact sural nerve in the SNI model; however, in contrast with PSL and SNL, in the SNI model the distal non-injured fibers are anatomically separated from injured fibers, protecting them from the inflammatory factors involved in the Wallerian degeneration. This may limit transcriptional changes in non-injured neurons in SNI compared to the other nerve ligation models.

In contrast to the non-injured neurons, the expression of hundreds of genes was altered in the injured neurons after SNI (Fig. 2B), some of which we confirmed by qRT-PCR and *in situ* hybridization (Fig. 2D and E). Previous genome-wide expression profiling studies using whole DRG tissue in the SNI and other neuropathic models have also demonstrated that nerve injury causes dramatic changes in myriad of genes^38^. However, our analysis of injured nociceptors revealed many novel genes and pathways that those previous studies did not detect (Fig. 3A). In particular, comparison of our microarray data of nociceptors with those of whole DRG tissue after SNI^1, 21^ revealed the enrichment of genes associated with oxidative stress and reactive oxygen species (ROS) (Fig. 3C). These include the mitochondrial superoxide dismutase SOD2, the metallothionein MT3 and CASP6, genes which expression were validated using qRT-PCR (Fig. 3E). Excessive ROS production was observed in animals models with tissue and nerve injury, and antioxidant treatments were analgesic in both inflammatory and neuropathic pain^39–47^. ROS are generated during mitochondrial oxidative metabolism and tightly control by antioxidants, such as SOD2^48^ and metallothioneins^49^. Interestingly, the absence metallothioneins from the injured peripheral nerves of patients with complex regional pain syndrome compared to control patients suggests a potential pathological role of these proteins, including MT3^50^. This may suggest a functional role for the observed and confirmed downregulation of MT3 in the damaged peripheral nerves in generating chronic pain.

The functional role of CASP6 in oxidative stress and pain is unclear. Here, we found that CASP6 mRNA and protein expression increases significantly in small-sized murine DRG neurons (i.e. nociceptors) after SNI (Fig. 4C and D). We have also demonstrated that CASP6 inhibition significantly reverse the development of mechanical allodynia (Fig. 4F) in line with our previous work showing that inhibition or silencing of CASP6 dramatically reduced symptoms of both inflammatory and neuropathic pain^23, 51^. Mechanistically, we have also demonstrated that peripheral tissue and nerve injury results in CASP6 release from axonal terminals, which then acts on microglial cells to trigger their activation and TNF-α release, inducing central sensitization and supporting the transition from acute pain to chronic pain^23^. Although we believe that this mechanism is a major driver in the traumatic animal models of chronic pain including in the SNI animal model, these results do not exclude the possibility of the contribution of CASP6 to other mechanism such as oxidative stress. Building on the insights afforded by our analysis, we hypothesized that in addition to pain arising from nerve injury, CASP6 might also contribute to chemotherapy-induced neuropathic pain (CIPN), since oxidative stress has been strongly implicated as an etiological factor for this form of pain^52^. Indeed, knock out of CASP6 attenuated mechanical allodynia in the paclitaxel model of chemotherapy induced neuropathic pain (CIPN) (Fig. 5B). In contrast to nerve injury, paclitaxel does not evoke microglial hypertrophy or proliferation in the spinal cord dorsal horn, suggesting that the mechanisms that produce neuropathic pain after exposure to chemotherapeutics may be fundamentally different than those operating after nerve injury^53^. Although identifying these mechanisms is not the scope of this research, CASP6 cleaves several proteins that control mitochondrial functions and oxidative stress. For instance, CASP6 can cleave and activate both CASP2 and CASP8 that are well-known to induce mitochondrial permeabilization^54^, as well as cleave and inactivate the antioxidant protein deglycase DJ-1, which leads to increase in the production of reactive oxygen species and oxidative stress in neuroblastoma cell lines^55^.

In conclusion, this study describes a reliable approach for the transcriptional analyses of homogenous cellular population of sensory neurons in neuropathic pain. Importantly, we have shown minor transcriptional changes in non-injured nociceptors after SNI surgery, suggesting that post-transcriptional processes may be the predominant drivers of functional plasticity of intact neurons in the neuropathic state. We have also revealed the transcriptional regulation of several new genes in injured nociceptors compared to previous whole DRG tissue analyses after SNI, which are mostly associated with the production of reactive oxygen species and oxidative stress. It is important to recognize that different neuronal changes and mechanisms may occur in various forms of chronic pain and evolve during the progression of neuropathic pain. However, we have validated our approach and results confirming the regulation of CASP6 in nociceptors after SNI and demonstrating its functional role in reducing neuropathic pain in animals after SNI and after paclitaxel injection, a well-characterized animal model associated with mitochondrial and oxidative stress. All together these data significantly increase our understanding of nociceptors and should provide a valuable resource for interpreting previous studies and draw new hypothesis for the treatment of neuropathic pain.

## Methods

### Animals and surgery

All the animal procedures were approved by the Institutional Animal Care and Use Committees of Duke University, Harvard Medical School and by the Canton of Vaud’s Committee on Animal Experimentation (Switzerland) and Duke University Institutional Animal Care and Use Committee in accordance with International Association for the Study of Pain guidelines^56^. The spared nerve injury (SNI) model of neuropathic pain was previously described in rats^31^. SNI was performed on adult male Sprague Dawley rats (between 300–350 g, Charles River France) by ligation and transection of tibial and common peroneal nerves leaving intact the sural nerve (Fig. 1A). To distinguish the cell soma in dorsal root ganglia corresponding to the injured and non-injured nerves a retrograde labelling approach based on our previously published studies^14, 16^. Briefly, a total 10 μl of Fast Blue retrograde tracer (FB, 2% in PBS, EMS-Chemie) were injected 4 days prior to SNI in the middle area of the hindpaw plantar surface to retrograde label cell soma from the injured tibial nerve, or in the lateral zone of the hindpaw plantar area just after surgery to retrograde label cell soma from the non-injured sural nerve (Fig. 1A). Instead of a single 10 μl FB bolus injection, multiple injections of 2 μl FB was used to limit the propagation of FB into unspecific area of the paw and labelling of non-injured nerves was performed after surgery to avoid the unintentional retrograde tracing of injured nerves. FB tracing has been previously used in association with transcriptional analysis^57^. Furthermore, although it is unlikely to affect mRNA expression, FB is present in both our control and experimental samples. Adult male CD1 mice (8–10 weeks, Charles River Laboratories) were used for immunohistochemistry and qRT-PCR assessment of CASP6 expression, and knockout mice lacking Casp6 (Casp6^−/−^, Stock No: 006236) and C57BL/6 background WT control mice were purchased from the Jackson Laboratory and used in behavioral assays. qRT-PCR and behavioral assays in mice were performed in an animal model of chemotherapy-induced neuropathic pain^58^ consisting in a single intraperitoneal injection of 6 mg/kg paclitaxel (Sigma-Aldrich).

### Behavior testing

Animals were habituated to the environment for at least 2 days before the testing. All the behaviors were tested blindly. For testing mechanical sensitivity, we confined mice in boxes placed on an elevated metal mesh floor and stimulated their hind paws with a series of von Frey hairs. For rats, hind paws were stimulated with a series of von Frey hairs with logarithmically increasing stiffness (0.04–26 g for rats and 0.02–2.56 g for mice, Stoelting), presented perpendicularly to the lateral plantar surface^31^ and behavioral results represent the 50% withdrawal threshold determined using the up and down method^59^. For mice, we tested paw withdrawal frequency in response to a subthreshold von Frey hair stimulation (0.4 g, 10 times). For testing cold allodynia, a drop of acetone was applied to the ventral surface of a hind paw and the mouse’s response was observed for 30 s after acetone application. Responses to acetone were graded according to the following 4-point scale: 0, no response; 1, quick withdrawal or flick of the paw; 2, prolonged withdrawal or flicking; 3, repeated flicking with licking. Acetone was applied alternately three times to each paw and the responses scored categorically^60^. The CASP6 inhibitor Z-V-E(OMe)-I-D(OMe)-FMK (Z-VEID-FMK) were purchased from R&D Systems (Minneapolis, MN) and intrathecal injections performed as previously described^23^.

### Laser capture microdissection (LCM)

Seven days after surgery, rats were decapitated after lethal intraperitoneal injection of pentobarbital sodium (100 mg/kg). L4/L5 DRGs were rapidly dissected (<10 min) in RNAse free environment, immediately embedded in O.C.T. compound (Sakura Finetek), placed at −20 °C for 1 h and then stored at −80 °C until cryosection. The cryosection was effectuated the same day of the microdissection. DRG tissue was sectioned at 10 μm and mounted on PALM POL-covered membrane slides (Zeiss). LCM was performed immediately after the sectioning using the Palm Robot-Microbeam system (Zeiss, Suppl. Video 1). Duration of LCM was limited to a maximum of 25 minutes per slide to preserve the RNA integrity. Samples were collected into microfuge caps moistened with a drop of mineral oil (Sigma-Aldrich), covered with 15 μl of Qiagen RTL buffer (Qiagen) with 1% β-mercaptoethanol (Sigma-Aldrich) and stored at −80 °C.

### Gene profiling

RNA from LCM samples was isolated using RNeasy Mini Kit (Qiagen, Basel, Switzerland), including a DNA digestion step. Before being used for a microarray, a small aliquot from each sample was subjected to a quality control test using the RNA 6000 Pico/Nano LabChip technology (Agilent). Only RNA with sharp and distinct 28 S and 18 S ribosomal RNA peaks and a ratio 28 S/18 S >1.7 or RIN^61^ >7 was further processed for microarray (Fig. 1D). cRNA double T7 linear amplification, was performed for each sample according to the Affymetrix standard protocol (GeneChip® Eukaryotic Small Sample Target Labeling Assay Version II, Affymetrix). A total of amplified and biotinylated cRNA was then processed on Affymetrix GeneChip Rat Genome 230 2.0 Array (GeneChip Expression Analysis, Technical Manual 701021, Affymetrix) and signal values and detection calls (present or absent) for all transcripts were assigned with the Affymetrix software MAS 5.0 (Affymetrix). Each cRNA samples processed on the array was obtained by the microdissection of DRG tissues from 6 rats. Nine arrays were processed for the gene profiling and a total of 56 rats were used for this experiment (6 rats, 3 conditions, and 3 duplicates). To identify differentially expressed transcripts, Mann–Whitney pairwise comparison analyses were carried out allowing the ranking of results by concordance of each identified change in gene expression. Genes for which the concordance in the pairwise comparisons exceeded the threshold of 77% (i.e. at least 7 of 9 array comparisons either increased or decreased) were considered as statistically significant. To limit the number of false positives, only genes that satisfied the pairwise comparison test and displayed ≥ |2|-fold change in expression were considered to be significantly regulated^62, 63^.

### Gene Ontology (GO) and Ingenuity Pathway Analysis (IPA)

To generate gene lists specific to the LCM methodology over a whole tissue analysis, we mined data from two studies using Affymetrix Array to profile transcriptional occurring in rat DRGs after SNI^1, 21^. Lists of genes unique to the LCM and whole tissue analysis (Suppl. Table 3) were questioned for GO term annotations by Database for Annotation, Visualization and Integrated Discovery (DAVID, http://david.abcc.ncifcrf.gov) and exclusive terms plotted as bars by their significance (Fig. 3B,C).

### Quantitative real time RT-PCR (qRT-PCR)

Ipsilateral L4/L5 DRGs from rats or mice were rapidly dissected, and frozen in RNA Later reagent (Qiagen). Total RNA was extracted using RNeasy Mini Kit (Qiagen, Basel, Switzerland) and RNA quality control tested using the RNA 6000 Nano LabChip technology (Agilent). RNA was then reverse transcribed using Omniscript reverse transcriptase or Quantitect RT Kit according to the manufacturer’s protocol (Qiagen). Quantitative real-time PCR was performed using SYBR-green qPCR with the MyiQ Single Color real-time PCR System or CFX96 Real-Time System (Bio-Rad, Reinach, Switzerland). Primer sets (Suppl. Table 4) were designed on OligoArchitect™ Online v3.0 (Sigma-Aldrich website). Specific PCR product amplification was confirmed using dissociation protocol and melting curve. All experiments were done at least in duplicate. Fold changes were determined using the relative standard curve method per the manufacturer’s instructions (Bio-Rad). Glyceraldehyde-3-phosphate dehydrogenase (GAPDH) was used as housekeeping gene since its expression in DRG is not altered by SNI surgery^64^.

### *In situ* hybridization (ISH)

SNI and sham rats were deeply anesthetized with sodium pentobarbital (100 mg/kg, i.p) and transcardially perfused with 0.9% saline followed by ice-cold 4% paraformaldehyde in 0.1 M phosphate buffer (PB, pH 7.4). L4 and L5 DRG tissues were dissected, post-fixed for 90 min in the same fixative and cryoprotected with 20% (w/v) sucrose in 0.1 M PB overnight at 4 °C. Tissues were sectioned to a thickness of 10 µm and mounted on slides. Sections were then processed for ISH using a digoxygenin-labeled antisense riboprobe. Riboprobe for activating transcription factor 3 transcripts (ATF3, NM_012912) was generated by PCR and T7 RNA polymerase transcription using the protocol described on Allen Brain Atlas (http://www.brain-map.org/). Riboprobe specificity was confirmed by DNA sequencing. We detected the hybridized riboprobe using anti-digoxygenin-alkaline phosphatase antibody (Roche, Basel, Switzerland). Sections were placed at 4 °C overnight and revealed using the NBT/BCIP/levamisole method (Roche, Basel, Switzerland). Fluorogold (FG) (2.5%; Fluorochrome) was injected (8 μl) in the sural skin territory of SNI-operated rats for retrograde tracing and identification of ATF3 positive neurons of non-injured sural nerve^14^.

### Immunohistochemistry (IHC)

Terminally anesthetized rats and mice were perfused with PBS, followed by 4% paraformaldehyde with 0.025% picric acid in PBS (PFA solution) and L4/L5 DRGs rapidly dissected. Post-mortem human lumbar DRG from a non-diseased donor (ND03910) was obtained via the National Disease Research Interchange program. All DRGs were post-fixed in PFA solution overnight and subsequently transferred into 20% sucrose in PBS for 24 h. Twelve-fifteen micrometer frozen sections were prepared, blocked for 1 h, and then incubated overnight at 4 °C in 1% BSA with 0.1% Triton X-100 in PBS with the following primary antibody: anti-peripherin (rabbit, 1:400, Chemicon), anti-neurofilament-200 (mouse, 1:300, Sigma-Aldrich), anti-substance P (SP, goat, 1:500, Santa Cruz Biotechnology), and anti-CASP6 (rabbit, 1:500, Cell Signaling Technology or rabbit, 1:1000, Abcam, Cambridge, UK). Primary antibodies were followed by Cy3- or FITC-conjugated secondary antibodies (1:400; Jackson ImmunoResearch Laboratories Inc., West Grove, PA) or FITC-conjugated IB4 (10 μg/ml; Sigma-Aldrich). NeuroTrace green fluorescent Nissl stain (Invitrogen, Carlsbad, CA) was used to reveal the morphology of the somata of neuronal cells. Sections were examined under a Zeiss or Nikon fluorescence microscope. The specificity of the CASP6 antibody was tested in our previous study^23^.

### Western Blot

Protein samples were prepared from dorsal root ganglia by homogenization in a lysis buffer containing protease and phosphatase inhibitors (Sigma-Aldrich), as previously described^23^. Samples were separated on SDS–PAGE gel and transferred to nitrocellulose blots. The blots were blocked and incubated overnight at 4 °C with antibodies against CASP6 (rabbit, 1:1000, Cell Signaling) and GAPDH (mouse, 1:20000, Millipore). These blots were incubated further with HRP-conjugated secondary antibody and developed in ECL solution. Specific bands were evaluated by apparent molecular sizes. The intensity of the selected bands was analyzed using NIH ImageJ software.

### Data analysis and availability

All data were expressed as mean ± SEM. Four to six animals per group were included for data analyses. Differences between groups were compared using Student’s t-test or ANOVA, followed by Tukey’s posttest or by unpaired, Student’s t test. The criterion for statistical significance was P < 0.05. Heat map and Pearson hierarchal clustering were generated by GENE-E (http://www.broadinstitute.org/cancer/software/GENE-E/). Data was plotted using Microsoft software (Excel) or Prism software (Graphpad). All data generated or analyzed during this study are included in this published article (and its Supplementary Information files) and raw data are available from the corresponding author on reasonable request.

## Electronic supplementary material

Suppl. Figures

Suppl. Video

Suppl. Table 1

Suppl. Table 2

Suppl. Table 3

Suppl. Table 4
