# Supplementary material for: Gene Expression Profiling of Cutaneous Injured and Non-Injured Nociceptors in SNI Animal Model of Neuropathic Pain
Source: Sci Rep. 2017 Aug 24;7:9367. doi: 10.1038/s41598-017-08865-3 (PMC5570923; doi:10.1038/s41598-017-08865-3)
Supplement: Supplementary file 1 — Suppl. Figures [file 41598_2017_8865_MOESM1_ESM.pdf]

GENE EXPRESSION PROFILING OF CUTANEOUS INJURED AND NON-INJURED NOCICEPTORS IN SNI ANIMAL MODEL OF NEUROPATHIC PAIN

Temugin Berta, Florence E. Perrin, Marie Pertin, Raquel Tonello, Yen-Chin Liu, Alex Chamessian, Ann C. Kato, Ru-Rong Ji, and Isabelle Decosterd

Supplementary Figures 1-3

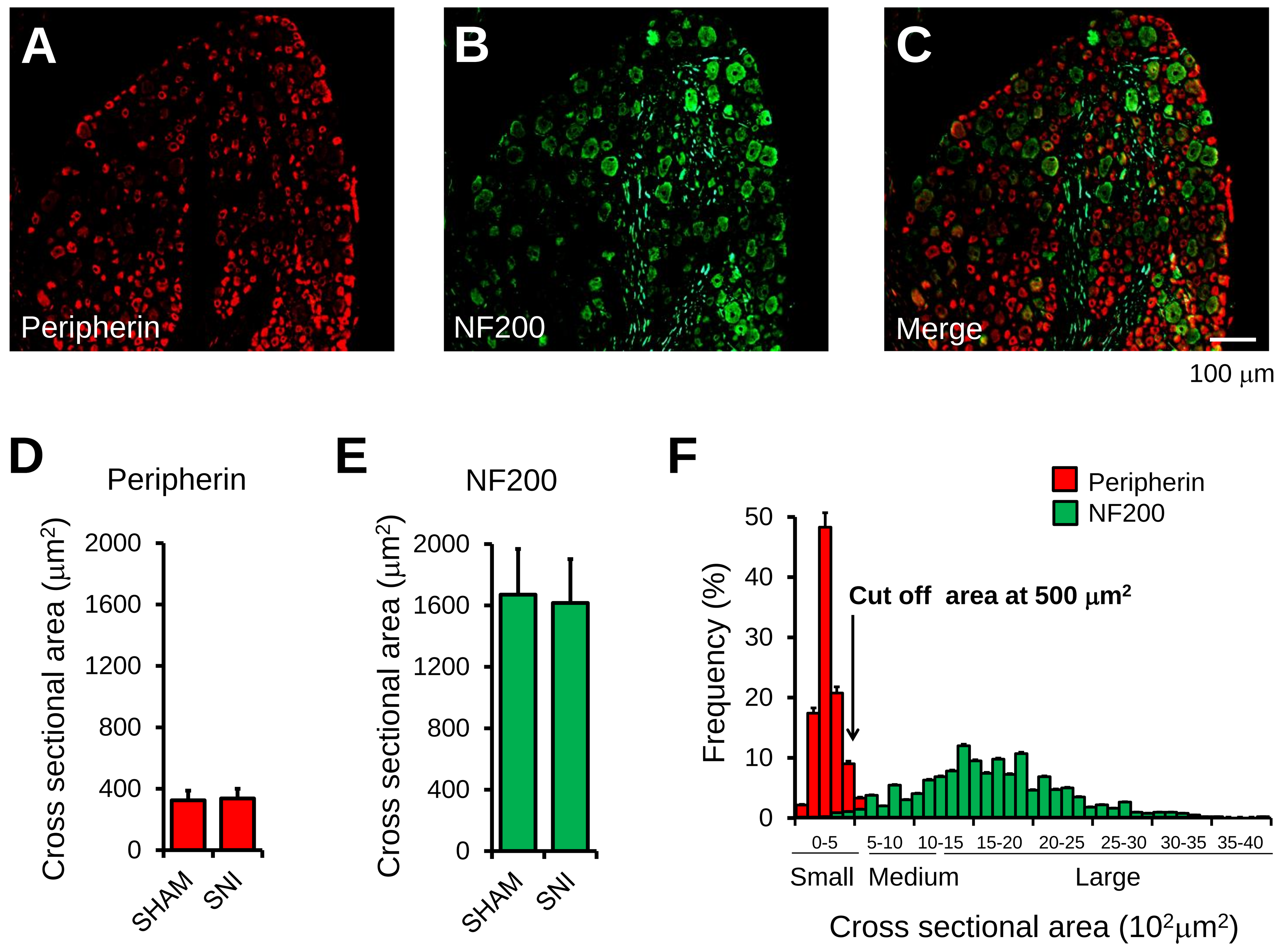

Supplementary Fig. 1: Definition of the cell size criteria for the LCM of nociceptors. Immunohistochemistry of fresh DRG tissue for **(A)** nociceptor marker peripherin, **(B)** large-sized neuron marker NF-200 and **(C)** merged figures (scale bars = 100 µm). Analysis of average cross-sectional area for **(D)** peripherin and **(E)** NF-200 in L4-L5 sections issued from 1 week SNI or sham animals. To note no significant differences were detected between the two surgeries. **(F)** Analysis of the cross-sectional area distribution for the peripherin- and NF-200-positive neurons reveal an optimal cut off cross-sectional area of 500 μm^2, within 90% of neurons were peripherin positive and only 5% were NF-200 positive.

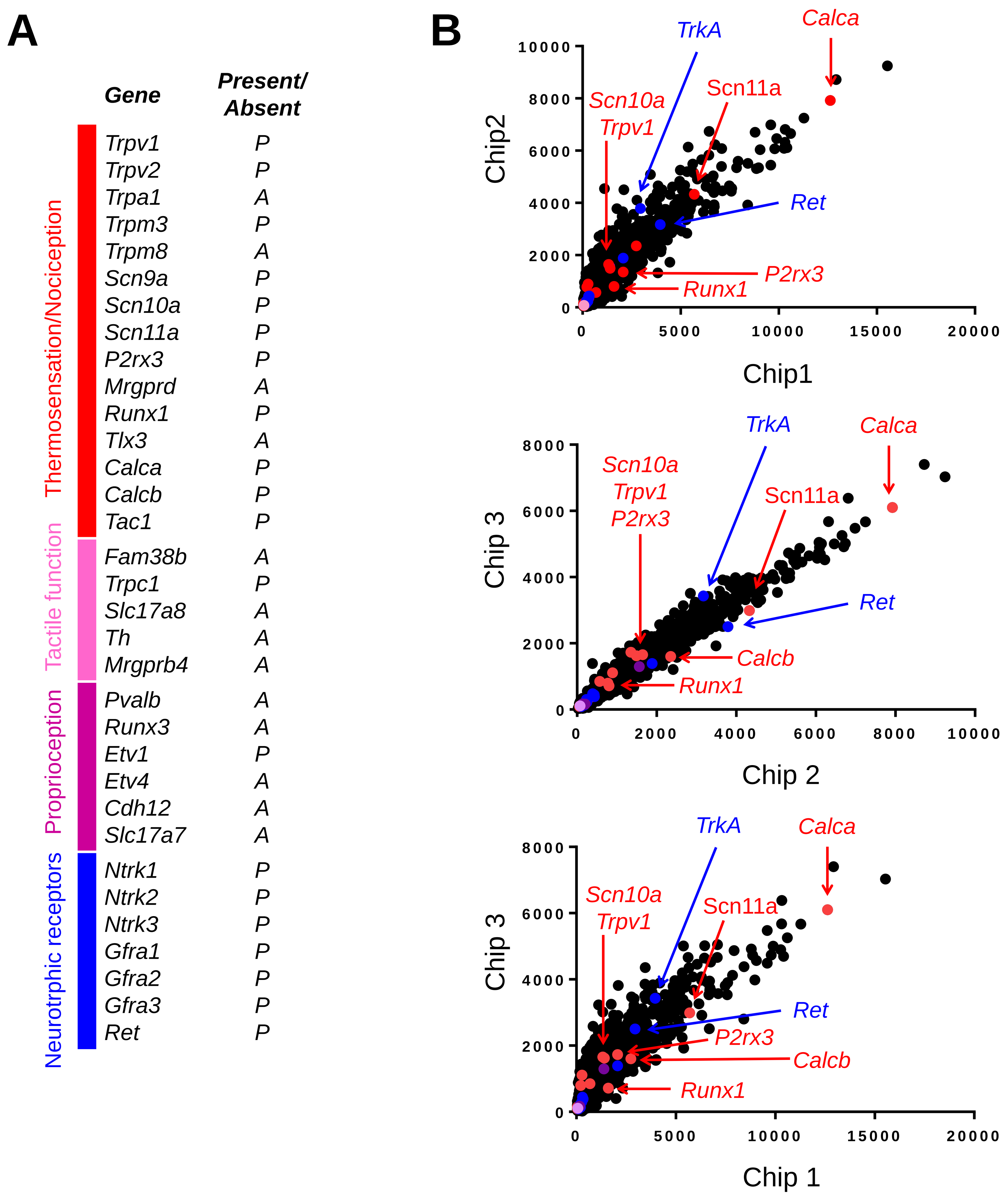

Supplementary Fig. 2: Expression of somatosensation markers on microarray analysis from LCM samples. **(A)** Analysis of transcript levels for known somatosensory mediators (list modified from Chiu *et al.* (ref)) in LCM samples from sham condition shows the presence of several markers for thermosensation and nociception. **(B)** Scatter plots of top 5% genes on the microarrays for the sham condition reveal the high expression of typical nociceptive markers, such as *Calca* (CGRP), *Scn11a* ($Na_v1.9$) and *Trpv1*.

Up-regulated genes

Fold	Non-injured	Injured
> 2	15	422
> 5	3	91
> 10	0	33

Down-regulated genes

Fold	Non-injured	Injured
> 2	6	180
> 5	0	7
> 10	0	0

Supplementary Fig. 3: Fold change of regulated gene. Number of up-regulated and down-regulated genes per conditions compared to sham and classified by fold change (for statistic see method section).
